# The epistatic relationship of *Drosophila melanogaster* CtIP and Rif1 in homology-directed repair of DNA double-strand breaks

**DOI:** 10.1093/g3journal/jkae210

**Published:** 2024-10-14

**Authors:** Makenzie S Thomas, Gautham S Pillai, Margaret A Butler, Joel Fernandez, Jeannine R LaRocque

**Affiliations:** Department of Human Science, School of Health, Georgetown University Medical Center, Washington, D.C. 20057, USA; Department of Human Science, School of Health, Georgetown University Medical Center, Washington, D.C. 20057, USA; Department of Human Science, School of Health, Georgetown University Medical Center, Washington, D.C. 20057, USA; Department of Human Science, School of Health, Georgetown University Medical Center, Washington, D.C. 20057, USA; Department of Human Science, School of Health, Georgetown University Medical Center, Washington, D.C. 20057, USA

**Keywords:** double-strand break repair, end-resection, homologous recombination, nonhomologous end-joining, single-strand annealing, CtIP, Rif1

## Abstract

Double-strand breaks (DSBs) are genotoxic DNA lesions that pose significant threats to genomic stability, necessitating precise and efficient repair mechanisms to prevent cell death or mutations. DSBs are repaired through nonhomologous end-joining (NHEJ) or homology-directed repair (HDR), which includes homologous recombination (HR) and single-strand annealing (SSA). CtIP and Rif1 are conserved proteins implicated in DSB repair pathway choice, possibly through redundant roles in promoting DNA end-resection required for HDR. Although the roles of these proteins have been well-established in other organisms, the role of Rif1 and its potential redundancies with CtIP in *Drosophila melanogaster* remain elusive. To examine the roles of DmCtIP and DmRif1 in DSB repair, this study employed the direct repeat of *white* (DR-*white*) assay, tracking across indels by decomposition (TIDE) analysis, and *P*{*wIw_2 kb 3*′} assay to track repair outcomes in HR, NHEJ, and SSA, respectively. These experiments were performed in *DmCtIP^Δ/Δ^* single mutants, *DmRif1*^*Δ/Δ*^ single mutants, and *DmRif1*^*Δ/Δ*^; *DmCtIP^Δ/Δ^* double mutants. This work demonstrates significant defects in both HR and SSA repair in *DmCtIP^Δ/Δ^* and *DmRif1*^*Δ/Δ*^ single mutants. However, experiments in *DmRif1*^*Δ/Δ*^; *DmCtIP^Δ/Δ^* double mutants reveal that DmCtIP is epistatic to DmRif1 in promoting HDR. Overall, this study concludes that DmRif1 and DmCtIP do not perform their activities in a redundant pathway, but rather DmCtIP is the main driver in promoting HR and SSA, most likely through its role in end resection.

## Introduction

Genome integrity, which is essential for cell survival and the accurate transmission of genetic information, is often threatened by the presence of DNA damage. Double-strand breaks (DSBs) are a particularly common and genotoxic form of DNA damage that occurs when both strands of the double-helix are broken as a result of endogenous or exogenous damage (Phillips and McKinnon 2007; Rodgers and McVey 2016). Failure to repair DSBs efficiently and accurately may result in chromosomal rearrangements, cell-death, or large-scale genomic instability that threatens cellular and organismal survival (van Gent *et al*. 2001; Phillips and McKinnon 2007; Rodgers and McVey 2016).

Several DNA repair pathways have evolved to maintain genome integrity and mitigate the consequences of unrepaired DSBs, including homologous recombination (HR), single-strand annealing (SSA), and nonhomologous end-joining (NHEJ). HR and SSA are homology-directed repair (HDR) pathways that require homologous sequences to direct repair (Janssen *et al*. 2016; Rodgers and McVey 2016). Both of these repair pathways are initiated by 5′ to 3′ end-resection, a critical step in HDR that occurs when endonucleases are recruited to the site of a DSB to cleave the DNA ends and form 3′ single-stranded overhangs.

SSA occurs when the DSB occurs between direct sequence repeats (>20 bp) with complementary base pairing. Once these complementary sequences are revealed through extensive end-resection, they may anneal to resolve the break, resulting in nucleotide deletions between direct repeats (Lieber *et al*. 2003; Symington and Gautier 2011). HR is the alternative homology-directed pathway that can occur following 5′ to 3′ end-resection. Once a homologous sequence is identified, 3′ single-strand overhangs will undergo RAD51-dependent strand invasion, resulting in the formation of a displacement loop (D-loop) that serves as the junction from which DNA repair can begin (San Filippo *et al*. 2008; Do *et al*. 2014). In contrast to HDR, NHEJ does not require extensive end resection. Rather, the two broken ends of the DNA molecule are recognized, may be minimally processed, and directly ligated, often resulting in small nucleotide insertions or deletions (indels) (Symington and Gautier 2011). As NHEJ and SSA involve the loss or gain of genetic information that may result in mutagenic alteration of sequences at the site of the break, they are considered error-prone repair pathways, while HR generally results in error-free repair (Rodgers and McVey 2016).

As end resection is a critical step in DSB repair pathway choice, there are several factors that drive this process. Mammalian CtBP (carboxy-terminal binding protein) interacting protein, or CtIP, serves as one of the most important regulators of end-resection in DSB repair due to its wide range of functions and crosstalk with various pathways. While CtIP has inherent endonuclease functions, specifically targeting 5′ ssDNA overhangs formed following the end-resection required for HDR to proceed, many studies demonstrate that the importance of CtIP in end-resection does not depend on its nuclease catalytic functioning (Makharashvili *et al*. 2014). Rather, CtIP is hypothesized to regulate DSB repair pathway choice through its interactions with other end-resection machinery, most notably the MRE11-RAD50-NBS11 (MRN) complex, BLM/DNA2, and BRCA1 (Wang *et al*. 2013; Anand *et al*. 2016). In *Drosophila melanogaster*, it has been shown that DmCtIP is a critical factor for HDR, as the loss of DmCtIP resulted in a significant 2-fold decrease in both HR and SSA repair (Yannuzzi *et al*. 2021). While DmCtIP is sufficient for HDR, its function in the context of other end-resection regulators is largely unknown.

Several additional end-resection factors may also contribute to DSB repair pathway choice. For example, *Rif1* (replication timing regulatory factor 1) is a conserved protein among eukaryotes with 92 homologs identified in organisms ranging from budding yeast to humans (Sreesankar *et al*. 2012). Though its evolutionarily conserved function may be to modulate chromatin structure via telomere associations or regulate replication timing, RIF1 has also been implicated in DSB repair pathway choice through its role in suppressing or promoting HDR (Sreesankar *et al*. 2012; Chapman *et al*. 2013). Specifically, mammalian RIF1 has emerged as a novel regulator of DNA end-resection and DSB repair pathway choice through its association with tumor suppressor, 53BP1 (Chapman, *et al*. 2013; Paiano *et al*. 2021). While it has been demonstrated that mammalian RIF1 inhibits end-resection, the *Saccharomyces cerevisiae* homolog displays opposing functions. Yeast Rif1 has been shown to promote DSB end-resection and increase HR repair by impeding the recruitment of the end-resection inhibitor, Rad9 (Martina *et al*. 2014). Despite the established function of *D. melanogaster* Rif1 in development (Sreesankar *et al*. 2015), its role in DSB repair pathway choice has yet to be characterized.

While DNA end-resection is a critical step in driving repair pathway choice between HDR and NHEJ, the roles of and relationship between DmCtIP and *Drosophila* Rif1 (DmRif1) are not fully understood. We find that both play a role in HR and SSA. However, they do not function redundantly and CtIP appears to have a more prominent role in HDR.

## Materials and methods

### Drosophila stocks and husbandry

*Drosophila* stocks and crosses were maintained on standard Nutrifly Bloomington Formulation medium (Genesee Scientific, San Diego, CA, USA) at 25°C with 12-hour light/dark cycles. DR-*white* transgenic stocks were previously described (Do *et al*. 2014). The Chromosome 2 P{*70I-SceI*} transgenic stocks contain an I-*Sce*I meganuclease transgene expressed by a *Drosophila heat-shock protein 70* (*hsp70*) promoter for heat shock induction (Rong and Golic 2000; Wei and Rong 2007). The *P*{*wIw_2 kb 3*′} SSA assay was a gift from the Sekelsky Lab (UNC-Chapel Hill) and described previously (Dewey *et al*. 2023). The *DmCtIP^Δ^* mutants were created by CRISPR/Cas9-mediated deletion of 1,653 base pairs in the *DmCtIP* coding region (Yannuzzi *et al*. 2021). The *DmRif1*^*1–7*^ (*DmRif1*^*Δ*^ herein) mutants, created by *P* element excision removing Exon 1 and the start site in the *DmRif1* coding region, were a gift from the McVey Lab (Tufts University). The DR-*white DmRif1*^*Δ*^ stock and the DR-*white DmRif1*^*Δ*^; *DmCtIP^Δ^* double mutant stocks were created through standard *Drosophila* genetics.

To establish the *DmRif1*^*Δ*^; *P*{*wIw_2 kb 3*′} *DmCtIP^Δ^* double mutant stock, *P*{*wIw_2 kb 3*′} and *DmCtIP^Δ^* were combined on Chromosome 3 as previously described (Yannuzzi *et al*. 2021). The *P*{*wIw_2 kb 3*′} assay and *DmCtIP^Δ^* on Chromosome 3 were then crossed into the *DmRif1*^*Δ*^ mutant background on Chromosome 2 using standard *Drosophila* genetics and confirmed by genotyping (see *DmCtIP* and *DmRif1* genotyping).

### *DmCtIP* and *DmRif1* genotyping

For genotyping, genomic DNA was isolated from individual flies using 50 µL Squishing Buffer (10 mM Tris-Cl, 25 mM NaCl, and 1 mM EDTA) and Proteinase K (0.2 mg/mL). Samples were incubated at 37°C for 30 min, followed by inactivation of Proteinase K at 95°C for 2 min. To genotype for *DmCtIP^Δ^* alleles, primers *DmCtIP*_-66f (5′-GGTCGGCTAACAAATACCAACC), and *DmCtIP*_1848a (5′-GGTCCCAAAACCGAGTGTCT) were used to screen for *DmCtIP* deletions with an expected PCR product of ∼284 bp. To genotype for *DmRif1*^*Δ*^ alleles, primers *Rif1*_-402f (forward, 5′-TTGCGAGTGTGGTGGTATCT) and *Rif1*_1500a (reverse, 5′-TGCGAATCCCTCAAAGTCCT) were used to screen for *DmRif1* deletions, with an expected PCR product of ∼1,000 bp. PCR was completed with SapphireAmp Fast PCR Master Mix (Clontech) with the following cycling conditions: 94°C, 3 min; [94°C, 30 s; 66°C touchdown (−0.5°C per cycle), 30 s; 72°C, 5 s] × 16; [94°C, 30 s; 58°C, 30 s; 72°C, 5 s] × 20; 72°C, 5 min and confirmed by gel electrophoresis (1% TAE agarose gel, 150Vh).

### DR-*white* assay

To analyze DSB repair pathway choice in *DmCtIP^Δ^* or *DmRif1*^*Δ*^ single mutants using the DR-*white* assay, *DmCtIP^Δ/Δ^*, *DmCtIP^Δ/+^*, *DmRif1*^*Δ/Δ*^, or *DmRif1*^*Δ/+*^ females containing DR-*white* were crossed to *DmCtIP^Δ/+^* or *DmRif1*^*Δ/+*^ males, respectively, containing the heat-inducible I-*Sce*I transgene. For *DmRif1*^*Δ*^; *DmCtIP^Δ^* double mutants, *DmRif1*^*Δ/Δ*^; *DmCtIP^Δ/Δ^* or *DmRif1*^*Δ/+*^; *DmCtIP^Δ/+^* females containing DR-*white* were crossed to *DmRif1*^*Δ/+*^; *DmCtIP^Δ/+^* males containing the heat-inducible I-*Sce*I transgene. For all experiments, flies were removed from crosses, and 0–3 day-old progeny were heat-shocked at 38°C for 1 hour. Once developed into adults, 0–4 day-old single F1 male progeny containing both DR-*white* and the I-*Sce*I transgene that were either heterozygous or homozygous for the mutation of interest were crossed to five tester *yw* females in vials to isolate individual DSB repair events from the male germline. F2 progeny from 38 to 70 individual F1 male germlines of each genotype were scored (∼20–150 progeny/germline). Up to three replicates were performed for each genotype and data points were combined.

### *P* **{*wIw_2 kb 3***′**} SSA assay**

To analyze SSA repair outcomes in *DmRif1*^*Δ*^ single mutants using the *P*{*wIw_2 kb 3*′} SSA assay, *DmRif1*^*Δ/Δ*^ or *DmRif1*^*Δ/+*^ females containing *P*{*wIw_2 kb 3*′} were crossed to *DmRif1*^*Δ/+*^ males containing the heat-inducible I-*Sce*I transgene. For *DmCtIP^Δ^* single mutants and *DmRif1*^*Δ*^; *DmCtIP^Δ^* double mutants using the *P*{*wIw_2 kb 3*′} SSA assay, *DmCtIP^Δ/Δ^*; *DmRif1*^*Δ/Δ*^ or *DmCtIP^Δ/+^*; *DmRif1*^*Δ/+*^ females containing *P*{*wIw_2 kb 3*′} were crossed to *DmRif1*^*Δ/+*^; *DmCtIP^Δ/+^* males containing the heat-inducible I-*Sce*I transgene. For all experiments, flies were removed from crosses, and 0–3 day-old progeny were heat-shocked at 38°C for 1 hour. 0–4 day old single F1 males, containing both *P*{*wIw_2 kb 3*′} and the I-*Sce*I transgene that were heterozygous for Dm*CtIP^Δ^* and/or *DmRif1*^*Δ*^ (*DmRif1*^*Δ/+*^; *DmCtIP^Δ/+^*), homozygous mutant for Dm*CtIP^Δ^* (*DmRif1*^*Δ/+*^; *DmCtIP^Δ/Δ^*), homozygous mutant for *DmRif1*^*Δ*^ (*DmRif1*^*Δ/Δ*^), or homozygous double mutants for *DmCtIP^Δ^* and *DmRif1*^*Δ*^ (*DmRif1*^*Δ/Δ*^; *DmCtIP^Δ/Δ^*) were crossed to five tester *yw* females in vials to isolate individual DSB repair events from the male germline. For each replicate, F2 progeny from 21 to 72 individual F1 male germlines of each genotype were scored (∼20–150 progeny/germline). Up to two replicates were performed for each genotype and data points were combined.

### Genomic DNA extraction

For tracking across indels by decomposition (TIDE) analysis, genomic DNA was extracted from F1 males and females carrying the DR-*white* assay as described (Sullivan *et al*. 2000). Single adult flies were homogenized in Buffer A (50 μL; 100 mM Tris-Cl pH 7.5, 100 mM EDTA, 100 mM sodium chloride, 0.5% SDS) and incubated at 65°C for 30 min. Buffer B (100 μL; 1.4 M potassium acetate, 4.3 M lithium chloride) was added and the mixture was incubated on ice for 30 min. Samples were centrifuged at 13,200 rpm for 15 min at 4°C. Supernatant was transferred to 100 μL of isopropanol and centrifuged at 13,200 rpm for 10 min at room temperature. The DNA pellet was washed with 250 μL of cold 70% ethanol, air-dried, and resuspended in 20 μL ddH_2_O (Sullivan *et al*. 2000).

### *Sce.white* polymerase chain reaction

For TIDE analysis, PCR reactions were performed on 100 ng of genomic DNA from single, whole fly samples in SapphireAmp Fast PCR Master Mix (Clontech). The *Sce.white* sequence was amplified using primers DR-*white*.8f (forward, 5′-GTGGATCAGGTAATCCAGG) and DR-*white*.8a (reverse, 5′-CTTAAGCCATCGTCAGTTGC) via the Touchdown 30 protocol as previously (Fernandez *et al*. 2019) with the following cycling conditions: 94°C, 3 min; [94°C, 30 s; 66°C touchdown (−0.5°C per cycle), 30 s; 72°C, 30 s] × 16; [94°C, 30 s; 58°C, 30 s; 72°C, 30 s] × 20; 72°C, 5 min. Samples were confirmed by gel electrophoresis (1% TAE agarose gel, 150Vh) and those with amplicons of 1.7 kb were column purified for Sanger Sequencing (see below).

### Column purification and TIDE analysis

PCR products were column purified using the Wizard SV Gel and PCR Clean-Up System (Promega) and eluted with 25 μL of nuclease-free water. 40–100 ng of purified DNA were prepared for sequencing using 25 pmol DR-*white*.9f primer (5′GAGCCCACCTCCGGACTGGAC3′). Samples were sequenced by Genewiz and analyzed by the TIDE algorithm, a computational protocol previously published and customized for the DR-*white* assay (Brinkman *et al*. 2014; Janssen *et al*. 2016; Fernandez *et al*. 2019). HR events were identified as restoration of the I-SceI recognition site to the wild-type SacI recognition sequence (GAGCTC), resulting in a 23 base pair deletion. Insertions and deletions of up to 35 base pairs were categorized as indels indicating a repair event using NHEJ with processing (NHEJ with indels hereafter). An output of zero base-pair indels was classified as the intact I-SceI recognition sequence, where no detectable repair events occurred. Sequencing chromatograms for each sample were analyzed for their quality, and sequences containing low background before the I-SceI recognition sequence were included in the dataset. The percentages of HR and NHEJ with indels calculated using TIDE were presented as a percentage of total detectable DSB repair events (HR + NHEJ with indels).

### Statistical analysis and graphical representation

Statistical tests are described within the Results section and were determined using GraphPad Prism (v. 10.1). Analysis of *DmCtIP^Δ^* single mutants, *DmRif1*^*Δ*^ single mutants, and *DmRif1*^*Δ*^; *DmCtIP^Δ^* double mutants were performed using a two-way ANOVA followed by Sidak's multiple comparisons test. Graphs were developed using GraphPad Prism (v. 10.1).

## Results

### DmRif1 is not redundant with DmCtIP in promoting repair by HR

**Fig. 1.**
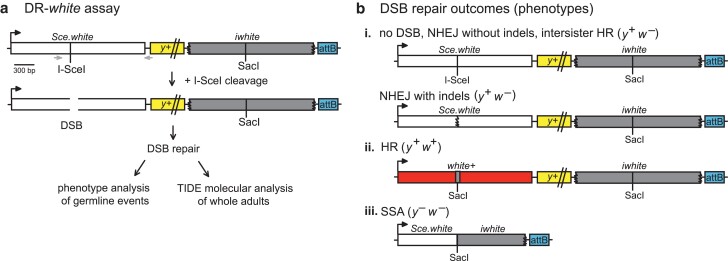
DR-*white* assay detects DSB repair events. a) The direct repeat of *white* (DR-*white*) assay contains two nonfunctional copies of the *white* gene: *Sce.white* (with a 23-bp insertion containing the I-SceI recognition site and introducing a premature stop codon) and *iwhite* (5′ and 3′ truncated donor sequence). Flies containing DR-*white* and the heat-shock-inducible I*-Sce*I transgene are heat shocked, resulting in I-SceI cleavage and a site-specific DSB that is subsequently repaired. F1 progeny display repair events occurring in the context of the whole organism, which can be analyzed by phenotype analysis of germline events or by molecular analysis of PCR across DSB events (arrows) via TIDE of whole adults. b) The resulting progeny of the DR-*white* assay are representative of single DSB repair events from the premeiotic germline: (i) white-eyed progeny (*y^+^w^−^*) are indicative of no DSB, intersister HR, or nonhomologous end-joining (NHEJ); (ii) red-eyed progeny (*y^+^w^+^*) indicate repair by intrachromosomal HR, as *white* gene function is restored by using the *iwhite* sequence as a donor; (iii) yellow-bodied, white-eyed progeny (*y^−^w^−^*) represent repair by SSA caused by deletion of the *y^+^* transgene when repetitive sequences are annealed.

The DR-*white* reporter assay allows for the quantification of intrachromosomal HR and SSA in the repair of a site-specific DSB (Do *et al*. 2014) (Fig. 1). The DR-*white* assay contains two nonfunctional copies of the *white* gene (Fig. 1a). The upstream *white* sequence, *Sce.white*, is nonfunctional due to the 23 base pair insertion of an I-SceI recognition site and premature stop codon. The downstream *white* sequence, *iwhite*, is nonfunctional due to truncations at the 5′ and 3′ ends. Repair of I-SceI-induced DSBs in the premeiotic germline can be captured by crossing heat-shocked males containing DR-*white* and I*-Sce*I to tester females. The progeny of this cross represent individual repair events from the male premeiotic germ line that can be distinguished phenotypically (Fig. 1b).

If there is no DSB formation, repair by NHEJ, or repair by intersister HR, the *Sce.white* sequence remains nonfunctional— resulting in brown-bodied, white-eyed (*y^+^w^−^*) progeny (Fig. 1b(i)). If repair by intrachromosomal noncrossover HR occurs, the I-SceI recognition sequence is converted to the wild-type SacI sequence from *iwhite* and *w+* expression is restored—resulting in brown-bodied, red-eyed (*y^+^w^+^*) progeny (Fig. 1b(ii)). If SSA occurs, the two *white* sequences are annealed after extensive (∼7.4 kb) resection and the intervening *y+* transgene is lost—resulting in yellow-bodied, white-eyed (*y^–^w^−^*) progeny (Fig. 1b(iii)). These phenotypes are scored to determine the frequencies of individual premeiotic germline DSB repair events.

**Fig. 2.**
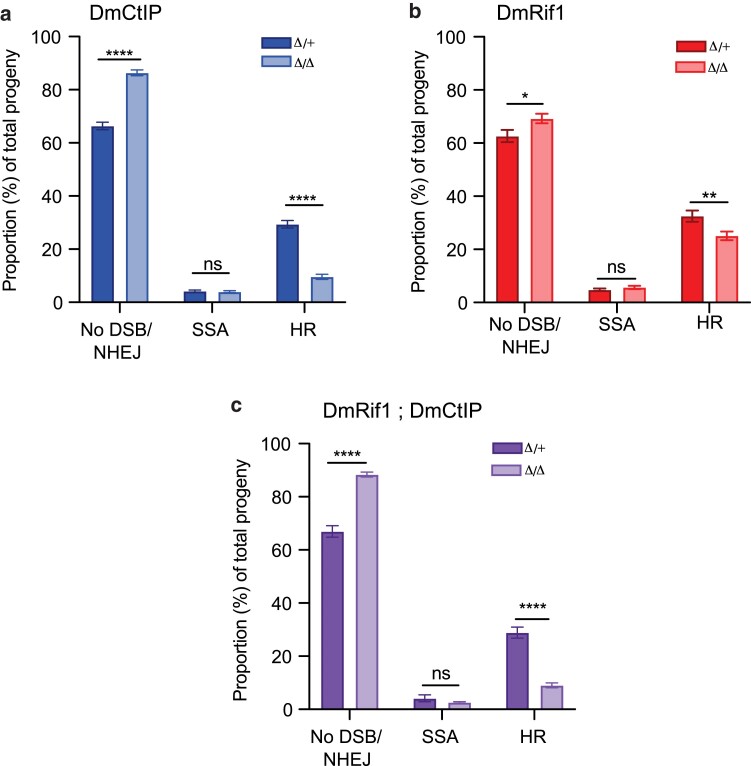
DSB repair pathway choice in the premeiotic germline using DR-*white* assay. F1 males of the indicated genotype containing the DR-*white* assay and I-*Sce*I transgene were crossed to *yw* tester females. The progeny were phenotypically analyzed to determine the proportion of no DSB/NHEJ, SSA, and HR repair. a) *DmCtIP^Δ/Δ^* mutants (*n* = 70 germlines) and heterozygous controls (*n* = 68 germlines). b) *DmRif1*^*Δ/Δ*^ mutants (*n* = 55 germlines) and heterozygous controls (*n* = 56 germlines). c) *DmRif1*^*Δ/Δ*^; *DmCtIP^Δ/Δ^* double mutants (*n* = 46 germlines) and heterozygous controls (*n* = 38 germlines). *P < 0.05, **P < 0.01, ****P < 0.0001 by two-way ANOVA followed by Sidak's multiple comparisons test. Bars represent means; error bars are SEM values. Data points available in Supplementary File 1.

To determine the influence of DmCtIP on DSB repair pathway choice, the DR-*white* assay was performed in *DmCtIP^Δ/Δ^* mutants and compared to heterozygous controls (*DmCtIP^Δ/+^*). The results of this experiment revealed an increase in the NHEJ/no DSB/intersister HR class, from 66.4 ± 1.4% in *DmCtIP^Δ/+^* heterozygous controls to 86.4 ± 1.1% in *DmCtIP^Δ/Δ^* mutants (*P* < 0.0001 by two-way ANOVA followed by Sidak's multiple comparisons test). Noncrossover HR events also decreased by ∼67%, from 29.0 ± 1.4% in *DmCtIP^Δ/+^* heterozygous controls to 9.6 ± 1.0% in *DmCtIP^Δ/Δ^* mutants (*P* < 0.0001 by two-way ANOVA followed by Sidak's multiple comparisons test) (Fig. 2a).

To determine the influence of DmRif1 on DSB repair pathway choice, the DR-*white* assay was performed in *DmRif1*^*Δ/Δ*^ mutants and compared to heterozygous controls (*DmRif1*^*Δ/+*^). Through phenotypic analysis of the DR-*white* assay, the proportions of DSB repair by HR, no DSB/NHEJ, and SSA in the premeiotic germline of *DmRif1*^*Δ/Δ*^ mutants were determined, revealing a significant decrease in noncrossover HR events and significant increase in the NHEJ/no DSB/intersister HR class in *DmRif1*^*Δ/Δ*^ mutants compared to controls. There was no difference in the proportion of SSA repair between groups (*P* > 0.05 by two-way ANOVA followed by Sidak's multiple comparisons test). The NHEJ/no DSB/intersister HR class increased from 62.7 ± 2.3% in *DmRif1*^*Δ/+*^ heterozygous controls to 69.2 ± 1.8% in *DmRif1*^*Δ/Δ*^ mutants (*P* < 0.05 by two-way ANOVA followed by Sidak's multiple comparisons test) (Fig. 2b). Notably, this assay revealed a ∼30% decrease in noncrossover HR events, from 32.5 ± 2.1% in *DmRif1*^*Δ/+*^ heterozygous controls to 25.1 ± 1.6% in *DmRif1*^*Δ/Δ*^ mutants (*P* < 0.01 by two-way ANOVA followed by Sidak's multiple comparisons test), thus revealing that, similar to DmCtIP, DmRif1 also promotes HR repair in premeiotic germline, although to a lesser extent.

The DR-*white* assay was repeated in *DmRif1*^*Δ/Δ*^; *DmCtIP^Δ/Δ^* double mutants to elucidate the potential synergistic contributions of DmCtIP and DmRif1 in DSB repair pathway choice. This assay revealed an increase in the NHEJ/no DSB/intersister HR class, from 67.0 ± 2.1% in *DmCtIP^Δ/+^*; *DmRif1*^*Δ/+*^ heterozygous controls to 88.4 ± 0.9% in *DmRif1*^*Δ/Δ*^; *DmCtIP^Δ/Δ^* double mutants (*P* < 0.0001 by two-way ANOVA followed by Sidak's multiple comparisons test) (Fig. 2c). There was a ∼70% decrease in HR repair events, from 28.9 ± 2.0% in *DmCtIP^Δ/+^*; *DmRif1*^*Δ/+*^ heterozygous controls to 9.0 ± 0.9% in *DmRif1*^*Δ/Δ*^; *DmCtIP^Δ/Δ^* double mutants (*P* < 0.0001 by two-way ANOVA followed by Sidak's multiple comparisons test), suggesting a significant defect in HR repair (Fig. 2c). The proportions of DSB repair in *DmRif1*^*Δ/Δ*^; *DmCtIP^Δ/Δ^* double mutants were compared to *DmCtIP^Δ/Δ^* single mutants to determine the contribution of DmCtIP to the observed defects in HR repair, revealing no significant difference (*P* > 0.05 by two-way ANOVA followed by Sidak's multiple comparisons test). This comparison suggests that loss of CtIP is the main driver for the HR defects observed in the *DmRif1*^*Δ/Δ*^; *DmCtIP^Δ/Δ^* double mutants.

### DmRif1 is not redundant with DmCtIP in inhibiting repair by NHEJ

As the DR-*white* assay is incapable of phenotypically distinguishing between no DSB, NHEJ, and intersister HR repair events, further molecular analyses were required to determine the impact of *DmCtIP^Δ/Δ^* and *DmRif1*^*Δ/Δ*^ single mutants and *DmRif1*^*Δ/Δ*^; *DmCtIP^Δ/Δ^* double mutants on DSB repair pathway choice, specifically in HR and NHEJ with indels. Due to the defects in HR observed in the DR-*white* assay, a compensatory increase in NHEJ repair was predicted. Thus, the established TIDE method was used to definitively quantify the proportions of HR and NHEJ events across whole adult tissues (Brinkman *et al*. 2014; Fernandez *et al*. 2019).

**Fig. 3.**
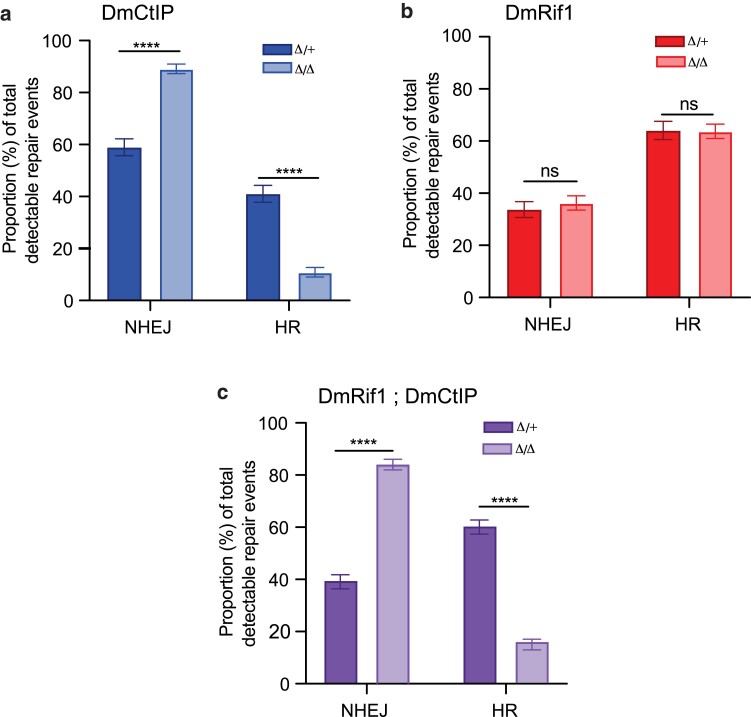
DSB repair pathway choice in whole-fly tissue using TIDE analysis. Whole flies of the indicated genotypes containing both the DR-*white* assay and the I-*Sce*I transgene were subject to molecular analysis by TIDE to determine relative proportions of NHEJ with indels and HR. a) *DmCtIP^Δ/Δ^* mutants (*n* = 49) and heterozygous controls (*n* = 49). b) *DmRif1*^*Δ/Δ*^ mutants (*n* = 34) and heterozygous controls (*n* = 33). c) *DmRif1*^*Δ/Δ*^; *DmCtIP^Δ/Δ^* double mutants (*n* = 20) and heterozygous controls (*n* = 31). ****P < 0.0001 by two-way ANOVA followed by Sidak's multiple comparisons test. Bars represent means; error bars are SEM values. Data points available in Supplementary File 1.

To determine the role of DmCtIP on DSB repair pathway choice, specifically NHEJ with indels, DNA sequences of individual *DmCtIP^Δ/Δ^* single mutant flies containing DR-*white* and I-*Sce*I-induced DSB repair events were analyzed. These results revealed a ∼1.5-fold increase in NHEJ with indels, from 59.0 ± 3.3% in *DmCtIP^Δ/+^* heterozygous controls to 89.1 ± 1.8% in *DmCtIP^Δ/Δ^* single mutants (*P* < 0.0001 by two-way ANOVA followed by Sidak's multiple comparisons test). There was a proportional decrease in HR repair events, from 41.0 ± 3.3% in *DmCtIP^Δ/+^* heterozygous controls to 10.9 ± 1.8% in *DmCtIP^Δ/Δ^* single mutants (*P* < 0.0001 by two-way ANOVA followed by Sidak's multiple comparisons test) (Fig. 3a). These results are consistent with what was previously described in *DmCtIP^Δ/Δ^* single mutants using the DR-*white* assay and supports the hypothesis that an inability to undergo end-resection will shift the cell toward error-prone NHEJ repair to resolve DSBs, leading to a decrease in observed HR.

To analyze the role of DmRif1 in repair by NHEJ with indels, DNA sequences of individual *DmRif1*^*Δ/Δ*^ single mutant flies containing DR-*white* and I-*Sce*I-induced DSB repair events were analyzed. This experiment found no significant difference in HR or NHEJ with indels repair events for *DmRif1*^*Δ/Δ*^ single mutants compared to *DmRif1*^*Δ/+*^ heterozygous controls (*P* > 0.05 by two-way ANOVA followed by Sidak's multiple comparisons test) (Fig. 3b). While the DR-*white* assay performed in *DmRif1*^*Δ/Δ*^ mutants reports 25.1 ± 1.6% of DSBs repaired through HR, the results of TIDE analysis report 63.7 ± 2.8% of DSBs repaired through HR. Additionally, the DR-*white* assay reports a significant defect in HR in *DmRif1*^*Δ/Δ*^ single mutants, while TIDE does not. These discrepancies are likely due to tissue and methodological differences in the assays, as DR-*white* measures DSB repair in the premeiotic germline through phenotypic analysis while TIDE measures DSB repair across all adult tissues through molecular analysis. Compared to the previous DR-*white* experiments performed in the premeiotic germline, these analyses suggest that the minor role of DmRif1 in promoting HR repair may be isolated to the premeiotic germline rather than whole adult tissues.

To investigate the combined contributions of DmCtIP and DmRif1 on HR vs NHEJ with indels repair, DNA sequences of individual *DmRif1*^*Δ/Δ*^; *DmCtIP^Δ/Δ^* double mutant flies containing DR-*white* and I-*Sce*I-induced DSB repair events were analyzed. These results revealed a ∼2-fold increase in NHEJ with indels, from 39.5 ± 2.7% in *DmCtIP^Δ/+^*; *DmRif1*^*Δ/+*^ heterozygous controls to 83.8 ± 2.0% in *DmRif1*^*Δ/Δ*^; *DmCtIP^Δ/Δ^* double mutants (*P* < 0.0001 by two-way ANOVA followed by Sidak's multiple comparisons test) (Fig. 3c). There was a proportional decrease in HR repair events, from 60.5 ± 2.7% in *DmRif1*^*Δ/+*^; *DmCtIP^Δ/+^* heterozygous controls to 16.2 ± 2.0% in *DmRif1*^*Δ/Δ*^; *DmCtIP^Δ/Δ^* double mutants (*P* < 0.0001 by two-way ANOVA followed by Sidak's multiple comparisons test) (Fig. 3c). The proportions of DSB repair in *DmRif1*^*Δ/Δ*^; *DmCtIP^Δ/Δ^* double mutants were compared to *DmCtIP^Δ/Δ^* single mutants to determine the contribution of DmCtIP to the observed differences in HR and NHEJ repair, revealing no significant difference (*P* > 0.05 by two-way ANOVA followed by Sidak's multiple comparisons test). This comparison once again suggests that loss of DmCtIP is the main driver for the HR defects observed in the *DmRif1*^*Δ/Δ*^; *DmCtIP^Δ/Δ^* double mutants, which is compensated by an increase in NHEJ repair.

### DmRif1 is not redundant with DmCtIP in SSA repair

Although the DR-*white* assay is capable of detecting SSA repair events, they are rare and may not accurately represent the true proportions of SSA that occur following induction of a DSB due to the extensive end-resection (∼7.4 kb) required to reveal and anneal repetitive sequences. As it has been previously demonstrated, the more extensive end-resection required to reveal complementary sequences for SSA, the more likely repair will go through an alternative homology-directed pathway, such as HR, in order to prevent mutagenic deletions of the intervening sequence (Fishman-Lobell *et al*. 1992). SSA repair efficiency is also heavily dependent upon the length of homology and length of end resection (Dewey *et al*. 2023). As end-resection is a two-step process that involves both short and long end-resection, the *P*{*wIw_2 kb 3*′} assay was employed to distinguish how DmRif1 and DmCtIP may differentially promote short end-resection for HR repair vs long end-resection for SSA repair as well as address the methodological deficiencies of the DR-*white* assay in detecting SSA events.

**Fig. 4.**
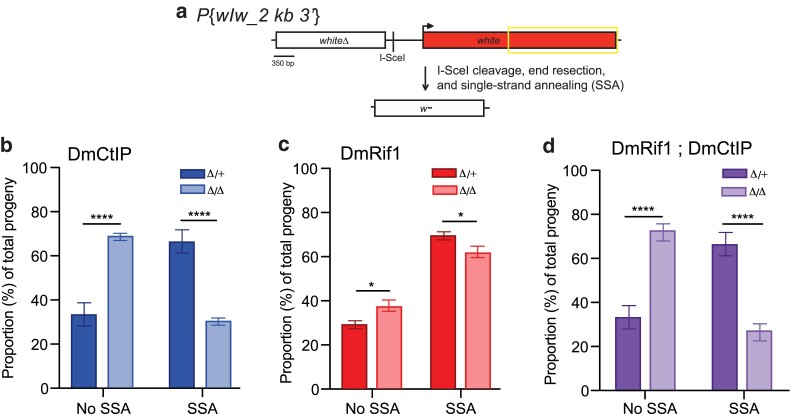
SSA repair in the premeiotic germline using *P*{*wIw_2 kb 3*′} assay. a) The *P*{*wIw_2 kb 3*′} assay contains a nonfunctional (*whiteΔ*) and functional (*white*) copy of *white* with an intervening I-SceI recognition sequence. Areas of sequence homology between the repeats are outlined (∼2.1 kb). When *P*{*wIw_2 kb 3*′} flies are crossed to flies containing a heat-shock-inducible I-*Sce*I transgene and heat shocked, DSBs are generated and repaired. End-resection of ∼3.6 kb and SSA results in a single nonfunctional copy of *white* (*w^−^*) and white-eyed progeny. b) *DmCtIP^Δ/Δ^* mutants (*n* = 21) and heterozygous controls (*n* = 21). c) *DmRif1*^*Δ/Δ*^ mutants (*n* = 72) and heterozygous control (*n* = 69). d) *DmRif1*^*Δ/Δ*^; *DmCtIP^Δ/Δ^* double mutants (*n* = 22) and heterozygous controls (*n* = 21). *P < 0.05, ****P < 0.0001 by two-way ANOVA followed by Sidak's multiple comparisons test. Bars represent means; error bars are SEM values. Data points available in Supplementary File 1.

The *P*{*wIw_2 kb 3*′} assay allows for the quantification of SSA in the repair of a site-specific DSB (Fig. 4a). *P*{*wIw_2 kb 3*′} consists of two tandem *white* genes with an intervening I-SceI recognition site (Dewey *et al*. 2023). The upstream *white* gene is nonfunctional due to deletion of the 5′ UTR, exons 1 through 3, and into the fourth exon (representing the first ∼1,550 base-pairs of the coding sequence), while the downstream *white* gene is functional (Dewey *et al*. 2023). I-SceI-induced DSBs repaired by SSA in the premeiotic germline can be captured by crossing males collected after heat-shock to tester females. The progeny of this cross represent individual repair events that can be distinguished phenotypically.

If sufficient end-resection occurs (∼3.6 kb) and reveals sequence complementarity between *white* repeats, the DNA strands can repair through homology-directed SSA, resulting in a partial, nonfunctional copy of the downstream *white* gene and conferring white eyes (*w^−^*) in the progeny (Dewey *et al*. 2023). If no DSB occurs or end-resection is insufficient, the downstream *white* gene will remain functional, resulting in red-eyed (*w+*) progeny. These phenotypes are scored to determine the frequencies of individual germline SSA repair events (Fig. 4a).

The *P*{*wIw_2 kb 3*′} assay was performed in *DmCtIP^Δ/Δ^* mutants and compared to heterozygous controls to investigate how DmCtIP influences SSA repair. This experiment revealed a ∼54% decrease in SSA repair, from 66.6 ± 5.3% in *DmRif1*^*Δ/+*^; *DmCtIP^Δ/+^* heterozygous controls to 30.7 ± 1.8% in *DmRif1*^*Δ/+*^; *DmCtIP^Δ/Δ^* mutants (*P* < 0.0001 by two-way ANOVA followed by Sidak's multiple comparisons test) (Fig. 4b). The defect in SSA in *DmCtIP^Δ/Δ^* single mutants is consistent with previous work (Yannuzzi *et al*. 2021) and mirrors the defects in HR observed in the DR-*white* assay, suggesting that loss of DmCtIP impacts the ability to undergo HDR, likely through its role in promoting end-resection.

To determine the influence of DmRif1 on SSA, the *P*{*wIw_2 kb 3*′} assay was performed in *DmRif1*^*Δ/Δ*^ mutants. SSA repair in *DmRif1*^*Δ/Δ*^ mutants was significantly decreased compared to *DmRif1*^*Δ/+*^ heterozygous controls, shifting from 70.2 ± 1.8% in the heterozygous controls to 62.2 ± 2.6% in *DmRif1*^*Δ/Δ*^ mutants (*P* < 0.05 by two-way ANOVA followed by Sidak's multiple comparisons test) (Fig. 4c). As similarly observed in the DR-*white* assay for HR repair, loss of DmRif1 also results in defects in the other HDR pathways, including SSA.

As both the *DmCtIP^Δ/Δ^* and *DmRif1*^*Δ/Δ*^ single mutants exhibited defects in SSA repair, it was essential to investigate the potentially redundant roles of DmCtIP and DmRif1 in SSA repair. The *P*{*wIw_2 kb 3*′} assay was performed in *DmRif1*^*Δ/Δ*^; *DmCtIP^Δ/Δ^* double mutants and compared to heterozygous controls. These results revealed a ∼50% decrease in SSA repair, from 66.6 ± 5.3% in *DmCtIP^Δ/+^*; *DmRif1*^*Δ/+*^ heterozygous controls to 29.4 ± 4.3% in *DmRif1*^*Δ/Δ*^; *DmCtIP^Δ/Δ^* double mutants (*P* < 0.0001 by two-way ANOVA followed by Sidak's multiple comparisons test) (Fig. 4d). Comparing the proportion of SSA in *DmCtIP^Δ/Δ^* single mutants to *DmRif1*^*Δ/Δ*^; *DmCtIP^Δ/Δ^* double mutants revealed no significant differences, suggesting that the loss of CtIP is the main driver of SSA defects observed in the double mutants. (*P* > 0.05 by two-way ANOVA followed by Sidak's multiple comparisons test). Building upon the previous results, this comparison further substantiates that DmCtIP is the main driver in promoting end-resection required to undergo HDR.

## Discussion

This study aimed to elucidate the roles of DmCtIP and DmRif1 in their proposed functions to promote end-resection required for HDR pathways, including HR and SSA. While DmRif1 plays a role in promoting HR and SSA in the premeiotic germline, its contribution is secondary to the role of DmCtIP. The *DmRif1*^*Δ/Δ*^; *DmCtIP^Δ/Δ^* double mutants also revealed a significant defect in HR and SSA repair; when these results were compared to the *DmCtIP^Δ/Δ^* single mutants alone, there was no significant difference in repair. This demonstrates that the previously established role of DmCtIP to promote HDR is epistatic to the role of DmRif1. This study serves as one of the first investigations into the role of *Drosophila* Rif1 in DSB repair pathway choice and the first study attempting to elucidate the potential redundancies of CtIP and Rif1 in promoting end-resection required for HDR.

When analyzing DSB repair pathway choice in *DmRif1*^*Δ/Δ*^ single mutants through the DR-*white*, TIDE, and *P*{*wIw_2 kb 3*′} assays, it was expected that the proportion of HR would be higher compared to heterozygous controls, since mammalian RIF1 has been demonstrated to inhibit end-resection and HR repair by interacting with 53BP1 and antagonizing the recruitment of BRCA1 to the site of DSBs (Chapman *et al*. 2013; Paiano *et al*. 2021). However, similar studies performed in *S. cerevisiae* have identified a contradictory role of Rifl to promote end-resection by blocking Rad9 switching (Martina *et al*. 2014). Despite the conflicting roles of Rif1 in both yeast and mammalian systems, this study reveals that DmRif1 plays a minor role in promoting HR in the premeiotic germline. Our observation of a role of DmRif1 in promoting HR, rather than inhibiting, may be attributed to the lack of a 53BP1 ortholog in *Drosophila*, as 53BP1 interaction is critical for inhibiting end resection and promotion NHEJ in mammalian cells (Chapman *et al*. 2013).

The minimal role of DmRif1 in HR is in contrast to what was observed in *DmCtIP* mutants, where significant defects in HDR were demonstrated in both the premeiotic germline and whole-fly tissues. While the *DmCtIP^Δ/Δ^* single and *DmRif1*^*Δ/Δ*^ single mutant experiments both reveal defects in HR and SSA, the greater magnitude of HDR repair defects observed in the *DmCtIP^Δ/Δ^* single mutants suggest that CtIP is the main driver in promoting end-resection required for HDR. One potential hypothesis that may underlie these reported differences could be differences in expression levels during various developmental stages. DmRif1 is expressed in relatively low amounts in adult tissues but is highly expressed in embryonic tissues (Brown *et al*. 2014; Sreesankar *et al*. 2015). The high level of expression in embryos follows a time-dependent gradient, with DmRif1 expression gradually decreasing from the larvae to pupa to adult stages (Brown *et al*. 2014). Alternatively, compared to the relative expression of DmRif1, DmCtIP exhibits low to moderate levels of expression in tissues across all age ranges (Brown *et al*. 2014). As experiments in this study include I-SceI expression in 0–3 day-old flies, analyses in more specific developmental time points may elucidate this possibility.

While these hypotheses may explain the discrepancy between the activity of DmRif1 in the premeiotic germline vs adult tissues, it is critical to consider the other canonical functions of DmRif1 as an important regulator of replication timing, replication fork protection, telomere lengths, and development (Hayano *et al*. 2012; Yamazaki *et al*. 2012; Sreesankar *et al*. 2015; Hafner *et al*. 2018). Future studies could consider developing separation-of-function mutants for DmRif1 that allow for the specific delineation of its roles in development, replication timing, telomere protection, and DSB repair.

The *P*{*wIw_2 kb 3*′} assay facilitated the discernment of distinct roles of DmRif1 and DmCtIP in facilitating short end-resection for efficient HR repair (minimum of 23 bp in this system), as opposed to the long (∼3.6 kb) end-resection required for revealing repetitive sequences essential for SSA repair. The results of this study found that DmRif1 plays a minimal role in promoting long end-resection required for SSA repair. Despite the utility of the *P*{*wIw_2 kb 3*′} assay, one limitation is the possibility that white-eyed progeny do not encapsulate true SSA repair events. Although rare in wild-type flies, a white-eyed event can also occur in cases of cryptic end-joining which includes repair events such as alternative end-joining (alt-EJ) or NHEJ (Dewey *et al*. 2023). If these repair events result in a partial deletion of the functional downstream *white* gene, then the white-eyed phenotype will result. This limitation may have resulted in an overestimation in the proportion of SSA in these experiments. This may be particularly true in DmCtIP and DmRif1 mutants if aborted HDR events are resolved as deletions. Additional analyses may disclose whether there are important functional differences in how DmCtIP or DmRif1 promote long end-resection required for SSA repair.

The evolutionary divergence of Rif1 is particularly relevant to this study, given its opposing functions in mammals and *S. cerevisiae*. Phylogenetic analysis of eukaryotic Rif1 sequences suggests that invertebrate Rif1 orthologs are more similar to vertebrate orthologs compared to fungal orthologs (Sreesankar *et al*. 2012). The functional divergence of Rif1 has been demonstrated between mammals and yeast, particularly in its role to tip the BRCA1-53BP1 axis toward error-prone NHEJ repair in mammals, while inhibiting Rad9 (53BP1 ortholog) to promote HR repair in yeast (Escribano-Diaz *et al*. 2013). This study suggests that despite the evolutionary and structural similarities to mammalian Rif1, DmRif1 may play a more similar functional role to that displayed in yeast to promote end-resection required for HDR. This may be attributed to the absence of canonical Rif1 binding partners such as Rap1, Rif2, and 53BP1 in *Drosophila* (Sreesankar *et al*. 2015).

In summary, this study has helped to uncover the role of DmRif1 in DSB repair pathway choice and its possible redundancies with DmCtIP in promoting end-resection required for HDR. These results reveal that DmCtIP promotes HR and SSA in the premeiotic germline epistatically to DmRif1. The functional divergence of DSB repair proteins across different evolutionary lineages is important to investigate as it allows for a more thorough understanding of DSB repair and the complex regulatory mechanisms in place to promote genome integrity in multicellular organisms.

## Supplementary Material

jkae210_Supplementary_Data

## Data Availability

Strains are available upon request. The authors affirm that all data necessary for confirming the conclusions of the article are present within the article, figures, and Supplementary Material. Supplementary Material includes all data points used in the figures (Supplementary File 1). Supplemental material available at G3 online.
